# Supplementary material for: Risk of cancer in patients with insomnia: Nationwide retrospective cohort study (2009–2018)
Source: PLoS One. 2023 Apr 21;18(4):e0284494. doi: 10.1371/journal.pone.0284494 (PMC10121030; doi:10.1371/journal.pone.0284494)
Supplement: S1 Table — (PDF) [file pone.0284494.s001.pdf]

Supplementary Table S1. Prevalence of ICD-10 code insomnia in this study population in the year 2009

	**Total**	**Male**	**Female**
Population	3982012	2198911	1783101
Total Insomnia	134068 (3.37)	48092 (2.19)	85976 (4.82)
F510	53602	19700	33904
G470	91474	32339	59133
Both	11008	3947	7061
