# Supplementary material for: Risk of cancer in patients with insomnia: Nationwide retrospective cohort study (2009–2018)
Source: PLoS One. 2023 Apr 21;18(4):e0284494. doi: 10.1371/journal.pone.0284494 (PMC10121030; doi:10.1371/journal.pone.0284494)
Supplement: S2 Table — (PDF) [file pone.0284494.s002.pdf]

S2 Table. Risk of cancers according to insomnia stratified by age group.

Cancer Type	Age Group				*P* for interaction
	20-39	40-59	60-79	≥80	
All Cancer	**1.310(1.154,1.487)**	**1.139(1.100,1.180)**	**0.939(0.916,0.963)**	0.991(0.907,1.083)	**<.0001**
Stomach	0.553(0.276,1.106)	1.024(0.925,1.132)	**0.903(0.848,0.962)**	1.027(0.825,1.277)	0.0660
Colorectal	1.346(0.927,1.954)	1.074(0.986,1.170)	**0.843(0.795,0.893)**	1.091(0.902,1.320)	**<.0001**
Liver	1.200(0.622,2.313)	**1.283(1.146,1.437)**	1.050(0.975,1.130)	0.912(0.699,1.190)	**0.0138**
Pancreatic	0.685(0.256,1.831)	**1.160(1.014,1.327)**	0.946(0.872,1.027)	0.985(0.724,1.340)	0.0706
Lung	**2.079(1.175,3.680)**	**1.354(1.212,1.513)**	**1.087(1.027,1.151)**	0.932(0.761,1.142)	**0.0002**
Thyroid	**1.218(1.015,1.462)**	**1.108(1.032,1.189)**	1.013(0.916,1.120)	**2.293(1.322,3.975)**	**0.0137**
Lymphoma	0.978(0.406,2.360)	1.103(0.873,1.393)	1.081(0.925,1.264)	1.159(0.646,2.078)	0.9895
Oral	1.715(0.639,4.603)	**1.487(1.148,1.927)**	0.990(0.807,1.215)	0.955(0.458,1.995)	0.0810
Esophagus	.	**1.504(1.062,2.131)**	1.13(0.931,1.372)	1.264(0.626,2.552)	0.5689
Gallbladder	.	**1.545(1.128,2.116)**	1.011(0.859,1.191)	0.760(0.419,1.378)	0.0732
Biliary	.	1.113(0.848,1.460)	1.086(0.96,1.229)	1.006(0.678,1.494)	0.9759
Laryngeal	.	1.529(0.966,2.418)	0.98(0.741,1.295)	1.356(0.469,3.922)	0.4256
Renal	0.987(0.409,2.380)	**1.276(1.032,1.578)**	1.16(0.994,1.354)	0.639(0.278,1.473)	0.4234
Bladder	1.932(0.617,6.046)	**1.340(1.052,1.707)**	0.936(0.825,1.063)	0.746(0.486,1.145)	**0.0210**
Nerves	0.866(0.278,2.697)	1.234(0.957,1.592)	**1.231(1.041,1.455)**	0.629(0.305,1.297)	0.3194
Multiple myeloma	2.913(0.715,11.874)	0.857(0.555,1.323)	**1.242(1.017,1.516)**	0.732(0.224,2.400)	0.2082
Leukemia	1.122(0.419,3.004)	1.135(0.843,1.529)	**1.256(1.051,1.503)**	1.379(0.702,2.711)	0.9233
Skin	2.964(0.726,12.093)	1.026(0.562,1.874)	0.805(0.552,1.174)	0.306(0.041,2.269)	0.2222
In men					
Prostate	.	**1.439(1.224,1.693)**	1.059(0.984,1.139)	1.184(0.915,1.533)	**0.0083**
Testicular	0.989(0.138,7.075)	0.916(0.226,3.712)	1.269(0.687,2.344)	4.071(0.369,44.879)	0.7589
In women					
Breast	0.870(0.611,1.240)	1.066(0.977,1.163)	**0.809(0.709,0.924)**	1.085(0.514,2.289)	**0.0072**
Cervical	0.957(0.496,1.848)	0.958(0.759,1.209)	0.964(0.770,1.206)	0.875(0.344,2.222)	0.9979
Ovarian	**1.854(1.110,3.096)**	0.937(0.763,1.149)	0.923(0.753,1.133)	0.713(0.283,1.792)	0.0753
Corpus	1.154(0.477,2.792)	0.986(0.775,1.256)	0.963(0.712,1.305)	2.992(0.922,9.715)	0.3230

Adjusted for sex, low income, smoking, alcohol consumption, diabetes, hypertension, dyslipidemia and body mass index. aHR, adjusted hazard ratio; CI, confidence interval. Bold style indicates statistical significance.
