# Supplementary material for: Risk of cancer in patients with insomnia: Nationwide retrospective cohort study (2009–2018)
Source: PLoS One. 2023 Apr 21;18(4):e0284494. doi: 10.1371/journal.pone.0284494 (PMC10121030; doi:10.1371/journal.pone.0284494)
Supplement: S3 Table — (PDF) [file pone.0284494.s003.pdf]

S3 Table. Hazard ratio of cancer incidence according to 'preexisting' and 'newly diagnosed' insomnia, defining 'preexisting' as having insomnia diagnosis 5 years before 2009 health checkup.

	Insomnia	N	Event	Duration	IR per 1000	aHR	*P*-value
All cancer	No	3847944	198667	31067930.79	6.3946	1(Ref.)	**0.0074**
	Existing	69574	5947	533251.62	11.1523	**0.965(0.940,0.990)**	
	Newly diagnosed	64494	4802	505877.43	9.4924	1.022(0.993,1.052)	
Stomach	No	3847944	31392	31615581.34	0.99293	1(Ref.)	**0.0010**
	Existing	69574	879	548629.31	1.60217	**0.884(0.827,0.946)**	
	Newly diagnosed	64494	711	518914.38	1.37017	0.956(0.887,1.030)	
Colorectal	No	3847944	37595	31598844.36	1.18976	1(Ref.)	**< .0001**
	Existing	69574	1066	548246.88	1.94438	**0.841(0.791,0.895)**	
	Newly diagnosed	64494	855	518508.5	1.64896	0.914(0.854,0.978)	
Liver	No	3847944	13957	31690559.31	0.44042	1(Ref.)	0.9684
	Existing	69574	397	550918.81	0.72061	0.992(0.897,1.097)	
	Newly diagnosed	64494	312	520655.86	0.59924	1.011(0.903,1.131)	
Pancreatic	No	3847944	15267	31692152.14	0.48173	1(Ref.)	0.8500
	Existing	69574	543	550808.44	0.98582	1.001(0.918,1.091)	
	Newly diagnosed	64494	404	520548.36	0.7761	1.029(0.932,1.137)	
Lung	No	3847944	25265	31677513.57	0.79757	1(Ref.)	**0.0416**
	Existing	69574	1012	549993	1.84002	**1.070(1.004,1.140)**	
	Newly diagnosed	64494	711	520121.95	1.36699	1.059(0.983,1.141)	
Breast	No	3847944	17577	31658108.87	0.55521	1(Ref.)	0.0995
	Existing	69574	432	550101.52	0.78531	**0.900(0.817,0.991)**	
	Newly diagnosed	64494	413	519672.49	0.79473	**0.996(0.904,1.099)**	
Cervical	No	3847944	3307	31711395.77	0.10428	1(Ref.)	0.0860
	Existing	69574	80	551462.78	0.14507	**0.788(0.630,0.985)**	
	Newly diagnosed	64494	90	520984.69	0.17275	1.072(0.869,1.323)	
Thyroid	No	3847944	32551	31573104.64	1.03097	1(Ref.)	**0.0384**
	Existing	69574	702	548243.36	1.28045	1.018(0.944,1.098)	
	Newly diagnosed	64494	695	517959.52	1.3418	**1.102(1.022,1.188)**	
Lymphoma	No	3847944	4660	31710782.54	0.14695	1(Ref.)	0.8272
	Existing	69574	142	551406.21	0.25752	1.048(0.885,1.240)	
	Newly diagnosed	64494	102	521084.69	0.19575	0.972(0.799,1.184)	
Ovarian	No	3847944	4027	31712301.22	0.12699	1(Ref.)	**0.0374**
	Existing	69574	98	551471.73	0.17771	**0.769(0.629,0.941)**	
	Newly diagnosed	64494	100	521032.67	0.19193	0.961(0.787,1.172)	
Oral	No	3847944	1224	31720569.89	0.038587	1(Ref.)	0.1103
	Existing	69574	33	551627.64	0.059823	0.882(0.623,1.251)	

	Newly diagnosed	64494	39	521196.57	0.074828	1.375(0.998,1.894)	
Esophagus	No	3847944	2511	31718352.9	0.07917	1(Ref.)	0.3564
	Existing	69574	72	551542.98	0.13054	0.910(0.719,1.153)	
	Newly diagnosed	64494	70	521190.3	0.13431	1.154(0.909,1.464)	
Gallbladder	No	3847944	2794	31719070.44	0.08809	1(Ref.)	0.4826
	Existing	69574	124	551540.55	0.22482	1.005(0.838,1.204)	
	Newly diagnosed	64494	95	521149.19	0.18229	1.135(0.924,1.393)	
Biliary	No	3847944	7326	31712641.83	0.23101	1(Ref.)	0.7619
	Existing	69574	293	551315.05	0.53146	0.988(0.878,1.111)	
	Newly diagnosed	64494	216	520993.92	0.41459	1.050(0.916,1.202)	
Laryngeal	No	3847944	1322	31719797.65	0.041677	1(Ref.)	0.7585
	Existing	69574	44	551598.94	0.079768	1.086(0.802,1.471)	
	Newly diagnosed	64494	34	521191.94	0.065235	1.098(0.780,1.545)	
Renal	No	3847944	5091	31707131.11	0.16056	1(Ref.)	0.1554
	Existing	69574	157	551234.72	0.28482	1.115(0.949,1.309)	
	Newly diagnosed	64494	125	520888.49	0.23997	1.141(0.955,1.364)	
Bladder	No	3847944	6076	31705022.22	0.19164	1(Ref.)	0.7515
	Existing	69574	223	551031.1	0.4047	1.034(0.903,1.183)	
	Newly diagnosed	64494	162	520846.76	0.31103	1.049(0.897,1.227)	
Nerves	No	3847944	3290	31716886.39	0.10373	1(Ref.)	**0.0420**
	Existing	69574	128	551443.07	0.23212	**1.255(1.050,1.501)**	
	Newly diagnosed	64494	82	521129.42	0.15735	1.055(0.847,1.315)	
Multiple myeloma	No	3847944	2951	31716355.18	0.09304	1(Ref.)	0.1489
	Existing	69574	86	551544.37	0.15593	0.908(0.731,1.127)	
	Newly diagnosed	64494	86	521117.62	0.16503	1.206(0.973,1.496)	
Leukemia	No	3847944	2992	31717768.13	0.09433	1(Ref.)	< **.0001**
	Existing	69574	108	551518.33	0.19582	**1.236(1.017,1.500)**	
	Newly diagnosed	64494	109	521099.72	0.20917	**1.615(1.333,1.957)**	
Skin	No	3847944	5535	31706196.19	0.17457	1(Ref.)	0.8099
	Existing	69574	247	550929.36	0.44833	1.042(0.916,1.185)	
	Newly diagnosed	64494	161	520770.48	0.30916	0.989(0.845,1.157)	
Prostate	No	3847944	16850	31672612.68	0.53201	1(Ref.)	**0.0117**
	Existing	69574	605	549773.12	1.10045	**1.103(1.016,1.197)**	
	Newly diagnosed	64494	441	519947.7	0.84816	1.098(0.999,1.207)	
Testicular	No	3847944	434	31722486.98	0.013681	1(Ref.)	0.6465
	Existing	69574	9	551702.2	0.016313	1.289(0.662,2.509)	
	Newly diagnosed	64494	5	521309.41	0.009591	0.782(0.323,1.893)	

Adjusted for sex, low income, smoking, alcohol consumption, diabetes, hypertension, dyslipidemia and body mass index. IR, incidence rate; aHR, adjusted hazard ratio; CI, confidence interval. Bold style indicates statistical significance.
