# Supplementary material for: Risk of cancer in patients with insomnia: Nationwide retrospective cohort study (2009–2018)
Source: PLoS One. 2023 Apr 21;18(4):e0284494. doi: 10.1371/journal.pone.0284494 (PMC10121030; doi:10.1371/journal.pone.0284494)
Supplement: S4 Table — (PDF) [file pone.0284494.s004.pdf]

S4 Table. Hazard ratio of cancer incidence according to 'preexisting' and 'newly diagnosed' insomnia, defining 'preexisting' as having insomnia diagnosis 3 years before 2009 health checkup.

	Insomnia	N	Event	Duration	IR per 1000	aHR	*P*-value
All cancer	No	3847944	198667	31067930.79	6.3946	1(Ref.)	**0.0213**
	Existing	64023	5490	490332.68	11.1965	**0.966(0.940,0.993)**	
	Newly diagnosed	70045	5259	548796.38	9.5828	1.016(0.988,1.044)	
Stomach	No	3847944	31392	31615581.34	0.99293	1(Ref.)	**0.0008**
	Existing	64023	808	504497.72	1.60159	**0.878(0.819,0.942)**	
	Newly diagnosed	70045	782	563045.97	1.38887	0.956(0.890,1.027)	
Colorectal	No	3847944	37595	31598844.36	1.18976	1(Ref.)	**<.0001**
	Existing	64023	993	504085.65	1.9699	**0.849(0.797,0.905)**	
	Newly diagnosed	70045	928	562669.72	1.64928	**0.898(0.842,0.959)**	
Liver	No	3847944	13957	31690559.31	0.44042	1(Ref.)	0.9884
	Existing	64023	369	506590.07	0.7284	0.995(0.896,1.104)	
	Newly diagnosed	70045	340	564984.59	0.60179	1.006(0.903,1.121)	
Pancreatic	No	3847944	15267	31692152.14	0.48173	1(Ref.)	0.7598
	Existing	64023	498	506510.56	0.9832	0.993(0.908,1.087)	
	Newly diagnosed	70045	449	564846.25	0.79491	1.035(0.942,1.137)	
Lung	No	3847944	25265	31677513.57	0.79757	1(Ref.)	**0.0425**
	Existing	64023	935	505763.13	1.84869	1.067(0.999,1.139)	
	Newly diagnosed	70045	788	564351.82	1.39629	1.064(0.991,1.142)	
Breast	No	3847944	17577	31658108.87	0.55521	1(Ref.)	0.1485
	Existing	64023	398	505858.49	0.78678	0.906(0.820,1.002)	
	Newly diagnosed	70045	447	563915.52	0.79267	0.982(0.894,1.079)	
Cervical	No	3847944	3307	31711395.77	0.10428	1(Ref.)	0.0626
	Existing	64023	71	507127.35	0.14	**0.763(0.602,0.967)**	
	Newly diagnosed	70045	99	565320.12	0.17512	1.070(0.875,1.308)	
Thyroid	No	3847944	32551	31573104.64	1.03097	1(Ref.)	**0.0480**
	Existing	64023	644	504142.85	1.27742	1.019(0.942,1.102)	
	Newly diagnosed	70045	753	562060.02	1.33971	**1.094(1.018,1.177)**	
Lymphoma	No	3847944	4660	31710782.54	0.14695	1(Ref.)	0.8545
	Existing	64023	131	507059.69	0.25835	1.047(0.878,1.247)	
	Newly diagnosed	70045	113	565431.21	0.19985	0.980(0.813,1.182)	
Ovarian	No	3847944	4027	31712301.22	0.12699	1(Ref.)	**0.0394**
	Existing	64023	89	507126.36	0.1755	**0.763(0.618,0.943)**	
	Newly diagnosed	70045	109	565378.04	0.19279	0.949(0.784,1.149)	
Oral	No	3847944	1224	31720569.89	0.038587	1(Ref.)	0.2693
	Existing	64023	32	507255.18	0.063085	0.926(0.650,1.319)	

	Newly diagnosed	70045	40	565569.03	0.070725	1.283(0.935,1.761)	
Esophagus	No	3847944	2511	31718352.9	0.07917	1(Ref.)	0.3181
	Existing	64023	66	507176.38	0.13013	0.896(0.700,1.146)	
	Newly diagnosed	70045	76	565556.9	0.13438	1.151(0.915,1.447)	
Gallbladder	No	3847944	2794	31719070.44	0.08809	1(Ref.)	0.6040
	Existing	64023	116	507178.66	0.22872	1.018(0.844,1.228)	
	Newly diagnosed	70045	103	565511.08	0.18214	1.105(0.907,1.347)	
Biliary	No	3847944	7326	31712641.83	0.23101	1(Ref.)	0.9570
	Existing	64023	277	506954.76	0.5464	1.009(0.894,1.139)	
	Newly diagnosed	70045	232	565354.21	0.41036	1.018(0.893,1.160)	
Laryngeal	No	3847944	1322	31719797.65	0.041677	1(Ref.)	0.6135
	Existing	64023	44	507225.19	0.086746	1.165(0.861,1.578)	
	Newly diagnosed	70045	34	565565.69	0.060117	1.009(0.717,1.420)	
Renal	No	3847944	5091	31707131.11	0.16056	1(Ref.)	0.1524
	Existing	64023	149	506876.81	0.29396	1.144(0.970,1.349)	
	Newly diagnosed	70045	133	565246.4	0.2353	1.108(0.932,1.317)	
Bladder	No	3847944	6076	31705022.22	0.19164	1(Ref.)	0.6391
	Existing	64023	203	506715.17	0.40062	1.012(0.879,1.165)	
	Newly diagnosed	70045	182	565162.7	0.32203	1.073(0.926,1.245)	
Nerves	No	3847944	3290	31716886.39	0.10373	1(Ref.)	0.0573
	Existing	64023	117	507092.25	0.23073	**1.244(1.032,1.499)**	
	Newly diagnosed	70045	93	565480.24	0.16446	1.086(0.883,1.335)	
Multiple myeloma	No	3847944	2951	31716355.18	0.09304	1(Ref.)	**0.0497**
	Existing	64023	75	507191.66	0.14787	0.858(0.681,1.081)	
	Newly diagnosed	70045	97	565470.32	0.17154	**1.234(1.007,1.512)**	
Leukemia	No	3847944	2992	31717768.13	0.09433	1(Ref.)	**<.0001**
	Existing	64023	99	507156.72	0.19521	**1.227(1.002,1.502)**	
	Newly diagnosed	70045	118	565461.32	0.20868	**1.590(1.321,1.913)**	
Skin	No	3847944	5535	31706196.19	0.17457	1(Ref.)	0.9224
	Existing	64023	222	506630.66	0.43819	1.014(0.886,1.161)	
	Newly diagnosed	70045	186	565069.18	0.32916	1.027(0.887,1.189)	
Prostate	No	3847944	16850	31672612.68	0.53201	1(Ref.)	**0.0098**
	Existing	64023	555	505580.05	1.09775	1.083(0.994,1.179)	
	Newly diagnosed	70045	491	564140.77	0.87035	**1.122(1.025,1.227)**	
Testicular	No	3847944	434	31722486.98	0.013681	1(Ref.)	0.4829
	Existing	64023	9	507328.45	0.01774	1.386(0.712,2.699)	
	Newly diagnosed	70045	5	565683.16	0.008839	0.727(0.300,1.759)	

Adjusted for sex, low income, smoking, alcohol consumption, diabetes, hypertension, dyslipidemia and body mass index. IR, incidence rate; aHR, adjusted hazard ratio; CI, confidence interval. Bold style indicates statistical significance.
