# Supplementary material for: Risk of cancer in patients with insomnia: Nationwide retrospective cohort study (2009–2018)
Source: PLoS One. 2023 Apr 21;18(4):e0284494. doi: 10.1371/journal.pone.0284494 (PMC10121030; doi:10.1371/journal.pone.0284494)
Supplement: S5 Table — (PDF) [file pone.0284494.s005.pdf]

S5 Table. Hazard ratio of cancer incidence according to 'preexisting' and 'newly diagnosed' insomnia, defining 'preexisting' as having insomnia diagnosis 1 year before 2009 health checkup.

	Insomnia	N	Event	Duration	IR per 1000	aHR	*P*-value
All cancer	No	3847944	198667	31067930.79	6.3946	1(Ref.)	0.1497
	Existing	49800	4334	380069.36	11.4032	0.971(0.942,1.001)	
	Newly diagnosed	84268	6415	659059.69	9.7336	1.003(0.979,1.029)	
Stomach	No	3847944	31392	31615581.34	0.99293	1(Ref.)	**0.0016**
	Existing	49800	647	391206.85	1.65386	**0.884(0.817,0.956)**	
	Newly diagnosed	84268	943	676336.85	1.39428	0.937(0.878,1.001)	
Colorectal	No	3847944	37595	31598844.36	1.18976	1(Ref.)	**<.0001**
	Existing	49800	797	390916.69	2.0388	**0.863(0.804,0.926)**	
	Newly diagnosed	84268	1124	675838.69	1.66312	**0.879(0.828,0.933)**	
Liver	No	3847944	13957	31690559.31	0.44042	1(Ref.)	0.9312
	Existing	49800	301	392954.53	0.76599	1.017(0.907,1.141)	
	Newly diagnosed	84268	408	678620.13	0.60122	0.988(0.895,1.091)	
Pancreatic	No	3847944	15267	31692152.14	0.48173	1(Ref.)	0.4251
	Existing	49800	383	392932.66	0.97472	0.965(0.871,1.069)	
	Newly diagnosed	84268	564	678424.15	0.83134	1.048(0.963,1.140)	
Lung	No	3847944	25265	31677513.57	0.79757	1(Ref.)	0.0417
	Existing	49800	749	392312.91	1.90919	1.070(0.995,1.151)	
	Newly diagnosed	84268	974	677802.05	1.437	1.062(0.996,1.132)	
Breast	No	3847944	17577	31658108.87	0.55521	1(Ref.)	0.2794
	Existing	49800	314	392372.81	0.80026	0.940(0.840,1.051)	
	Newly diagnosed	84268	531	677401.2	0.78388	0.948(0.869,1.034)	
Cervical	No	3847944	3307	31711395.77	0.10428	1(Ref.)	0.1067
	Existing	49800	53	393404.15	0.13472	**0.747(0.569,0.981)**	
	Newly diagnosed	84268	117	679043.32	0.1723	1.021(0.849,1.229)	
Thyroid	No	3847944	32551	31573104.64	1.03097	1(Ref.)	**0.0419**
	Existing	49800	487	391145.64	1.24506	1.005(0.919,1.100)	
	Newly diagnosed	84268	910	675057.23	1.34803	**1.089(1.019,1.164)**	
Lymphoma	No	3847944	4660	31710782.54	0.14695	1(Ref.)	0.8381
	Existing	49800	96	393363.06	0.24405	0.972(0.793,1.191)	
	Newly diagnosed	84268	148	679127.84	0.21793	1.044(0.886,1.231)	
Ovarian	No	3847944	4027	31712301.22	0.12699	1(Ref.)	0.0912
	Existing	49800	72	393387.02	0.18303	0.809(0.640,1.023)	
	Newly diagnosed	84268	126	679117.38	0.18553	0.885(0.740,1.057)	
Oral	No	3847944	1224	31720569.89	0.038587	1(Ref.)	0.5700
	Existing	49800	27	393493.38	0.068616	0.985(0.671,1.447)	

	Newly diagnosed	84268	45	679330.84	0.066242	1.175(0.871,1.585)	
Esophagus	No	3847944	2511	31718352.9	0.07917	1(Ref.)	0.7359
	Existing	49800	56	393421.08	0.14234	0.942(0.721,1.229)	
	Newly diagnosed	84268	86	679312.19	0.1266	1.072(0.864,1.330)	
gallbladder	No	3847944	2794	31719070.44	0.08809	1(Ref.)	0.2148
	Existing	49800	84	393438.08	0.2135	0.932(0.750,1.160)	
	Newly diagnosed	84268	135	679251.67	0.19875	1.153(0.969,1.372)	
Biliary	No	3847944	7326	31712641.83	0.23101	1(Ref.)	0.9564
	Existing	49800	222	393239.24	0.56454	1.018(0.890,1.165)	
	Newly diagnosed	84268	287	679069.73	0.42264	1.009(0.896,1.136)	
Laryngeal	No	3847944	1322	31719797.65	0.041677	1(Ref.)	0.6354
	Existing	49800	36	393453.61	0.091497	1.174(0.841,1.638)	
	Newly diagnosed	84268	42	679337.27	0.061825	1.030(0.756,1.402)	
Renal	No	3847944	5091	31707131.11	0.16056	1(Ref.)	0.0886
	Existing	49800	125	393150.59	0.31794	**1.209(1.011,1.446)**	
	Newly diagnosed	84268	157	678972.62	0.23123	1.069(0.911,1.254)	
Bladder	No	3847944	6076	31705022.22	0.19164	1(Ref.)	0.7417
	Existing	49800	166	393034.18	0.42236	1.027(0.879,1.200)	
	Newly diagnosed	84268	219	678843.68	0.32261	1.050(0.917,1.203)	
Nerves	No	3847944	3290	31716886.39	0.10373	1(Ref.)	0.0609
	Existing	49800	93	393368.08	0.23642	**1.257(1.021,1.548)**	
	Newly diagnosed	84268	117	679204.41	0.17226	1.106(0.919,1.332)	
Multiple myeloma	No	3847944	2951	31716355.18	0.09304	1(Ref.)	0.2443
	Existing	49800	61	393438.45	0.15504	0.887(0.687,1.145)	
	Newly diagnosed	84268	111	679223.54	0.16342	1.142(0.944,1.382)	
Leukemia	No	3847944	2992	31717768.13	0.09433	1(Ref.)	**< .0001**
	Existing	49800	73	393428.96	0.18555	1.147(0.907,1.449)	
	Newly diagnosed	84268	144	679189.08	0.21202	**1.579(1.334,1.869)**	
Skin	No	3847944	5535	31706196.19	0.17457	1(Ref.)	0.6591
	Existing	49800	168	393023.84	0.42745	0.972(0.833,1.134)	
	Newly diagnosed	84268	240	678676	0.35363	1.056(0.927,1.202)	
Prostate	No	3847944	16850	31672612.68	0.53201	1(Ref.)	**0.0111**
	Existing	49800	465	392092.08	1.18595	**1.113(1.014,1.221)**	
	Newly diagnosed	84268	581	677628.74	0.8574	**1.091(1.004,1.186)**	
Testicular	No	3847944	434	31722486.98	0.013681	1(Ref.)	0.3828
	Existing	49800	8	393545.3	0.020328	1.518(0.750,3.074)	
	Newly diagnosed	84268	6	679466.31	0.00883	0.741(0.330,1.662)	

Adjusted for sex, low income, smoking, alcohol consumption, diabetes, hypertension, dyslipidemia and body mass index. IR, incidence rate; aHR, adjusted hazard ratio; CI, confidence interval. Bold style indicates statistical significance.
