# Supplementary material for: Risk of cancer in patients with insomnia: Nationwide retrospective cohort study (2009–2018)
Source: PLoS One. 2023 Apr 21;18(4):e0284494. doi: 10.1371/journal.pone.0284494 (PMC10121030; doi:10.1371/journal.pone.0284494)
Supplement: Attachment — Submitted filename: Response to Reviewers.docx [file pone.0284494.s006.docx]

March 2, 2023

Dear Editor:

We wish to resubmit the manuscript titled, "Risk of Cancer in Patients with Insomnia: Nationwide Retrospective Cohort Study (2009-2018)”. The manuscript ID is PONE-D-22-33830. The manuscript is coauthored by Kichul Yoon, Jin Hyung Jung, Eun Hyo Jin, Joo Hyun Lim, Seung Joo Kang, Yoon Jin Choi, and Dong Ho Lee.

In addition to the findings of our original manuscript, we further analyzed our data in terms of age groups and found that insomnia increased the risk of any cancer in younger age groups (20-39y and 40-59y) but it significantly decreased the risk in the 60-79y age group. Revised version of our manuscript included the novel results.

We thank you and the reviewers for your thoughtful suggestions and insights. The manuscript has benefited from these insightful suggestions. We look forward to working with you and the reviewers to move this manuscript closer to publication in the *PLOS ONE*.

All comments by the reviewers have been addressed in the revised manuscript. We believe these comments substantially improved our manuscript. Changes to the revised manuscript, including in the tables and in the figure legends, have been highlighted using RED font to avoid any confusion.

This manuscript has not been published or presented elsewhere in part or in entirety and is not under consideration by another journal. All study participants provided informed consent, and the study design was approved by the appropriate ethics review board. We have read and understood your journal’s policies, and we believe that neither the manuscript nor the study violates any of these. There are no conflicts of interest to declare.

Thank you very much for allowing us an opportunity to revise our manuscript. We look forward to hearing from you.

Sincerely,

Cheol Min Shin, M.D., Ph.D.

Kyungdo Han, Ph.D.

**Reviewer #1**

• This is a retrospective cohort study from the Korean National Health Insurance (KNHIS) database seeking to understand a potential link between the incidence of insomnia and risk of cancer diagnoses in this population.

• Adults (≥ 20 years old) enrolled in the KNHIS got standard exams every 2 years, which included lifestyle questionnaires and labs.

• Cohort selected 40% of subjects from 2009, followed over time until 2018. Previous cancer diagnoses excluded. Missing data excluded.

• Disease diagnoses classified by ICD-10 coding (including insomnia and cancers). Insomnia diagnoses stratified by incident or prevalent and timing. Cancer diagnoses included combined ICD-10 and insurance claim codes.

• Analyses for Hazard adjusted for age, sex, income, smoking, EtOH, Dm-2, HTN, HLD, BMI.

• 3.98M subjects included in analysis. Cancer diagnosis in older subjects (56.8 vs. 48.3 years). No difference in sex between CA and non-CA diagnoses. CA diagnoses were higher waist circumference, metabolic syndrome (DM-2, HTN, HLD), lower income, higher insomnia, smoking, alcohol use, BMI, etc.

• Per specific cancers, insomnia group had higher rates of certain cancers (i.e. colorectal, leukemia), and lower in others (ovarian, stomach), which was different by gender as well. No difference in cancer diagnoses rates by type of insomnia (existing, newly diagnosed, etc.).

• Summary – The authors present intriguing data regarding a potential association between a diagnosis of insomnia and the risk of developing cancer diagnoses. Strengths include a large and relatively comprehensive dataset utilizing the resources of a national healthcare system in Korea, and a robust analysis.

**Comments**

**1. The authors attempt to explain the lower rate of certain malignancies in insomnia patients (Discussion second paragraph), noting that insomniac patients may have higher rates of screening uptake for malignancy like colonoscopy. However, they go on to suggest the stomach cancer rate is potentially related to *H. pylori* infection, however I fail to see how this is linked to insomnia, and I would remove that potential explanation (one could posit any number of comparable explanations, like they consume more food with nitrates that is linked to stomach cancer, but explanations to link insomnia to lower malignancy rates should require further justification).**

***Answer:*** Thank you for your important comments. As you noted, we could not find any reference showing an association between insomnia and *H. pylori.* According to your recommendation, we revised the relevant sentences in the Discussion as follows (line numbers are of 'Clean Copy' version of manuscript;)(lines 253-257) “… To our knowledge, no researchers have reported a link between the important risk factor *Helicobacter pylori* status and insomnia. Other causes of stomach cancer, such as red meat or high salt intake, could also be considered. (18, 19) As an association between stress, dietary habit, and insomnia has been suggested, further investigation on dietary patterns of insomnia patients is needed to clarify this issue.(20) …”

In addition, as women showed a higher incidence of diffuse type gastric cancer, which is difficult to detect *via* screening endoscopy, it could also affect the sex difference we discovered in that only male insomnia patients had a lower risk of stomach cancer. We added this in the Discussion section as follows (line 250): “… In addition, the incidence of diffuse type gastric cancer, which we could not differentiate in the current data, is higher in young women than in men. As it is known that diffuse type gastric cancer is difficult to detect *via* screening endoscopy, it may have influenced the sex difference we found in our analysis.(17) …”

**2. In the fourth discussion paragraph, the third sentence should probably indicate the authors explaining a link between insomnia and oral cancer in females (“…insomnia could independently be a risk factor for oral cancer in female patients.”)**

***Answer:*** Thank you for your accurate comments. We revised the sentence as follows (line 270): “… Risk factors for oral cancers include smoking and heavy alcohol drinking. Both smoking and drinking are more prevalent in men, whereas these may play a minor role in female oral cancers. After adjusting for smoking and drinking, insomnia remains an independent risk factor for oral cancer in female patients but not in male patients. …”

**3. In the fifth discussion paragraph, I would not report the hazard from the cited reference itself, the reference is already provided. Additionally, the following sentence (the third sentence) should read “…there was a higher risk of nervous system cancer in female patients”. The following sentence should indicate the cancers themselves as well, “However, we could not independently** **validate this association as we did not collect data on peripheral and nervous system cancers specifically.**

***Answer:*** Thank you for your important comments. We removed the hazard ratios from the sentence. We revised the following sentences as follows (line 279): “… We found that there was a higher risk of nervous system cancer in female patients. However, we could not independently assess this association as data for peripheral and nervous system cancers could not be collected separately. ...”

**4. In the 8^th^ discussion paragraph, discussing the stratification of insomnia and risk of cancer, the authors suggest the existing insomnia (within 5 years) is not long enough to fully evaluate the risk of cancer. This is a theory, not a fact, and should be treated as such, “…multiple sequential mutations from the exposure of possible causes (19), the 5-year duration may not be long enough to evaluate the risk of cancer development.”**

***Answer:*** Thank you for pointing this out. We revised the paragraph as follows (line 296): “… Generally, as cancer development requires a substantial amount of time for multiple sequential mutations from the exposure of possible causes,(19) the 5-year duration may not be long enough to evaluate the risk of cancer development. …”

**5. In the 9th discussion paragraph, the link between insomnia and lower cancer rates, secondary potentially to medical-care-seeking behavior, should be rephrased. (“…attributed to insomnia associated with personality traits such as hypochondriasis or anxiety, may inadvertently promote early detection of premalignant lesions…”).**

***Answer:*** Thank you for your important comments. We revised the paragraph as follows (lines 305-309): “… Our result regarding unexpected lower risk of several cancers including colon (both sexes), stomach (men), and ovarian malignancy might be attributed to insomnia associated with personality traits such as hypochondriasis or anxiety and may inadvertently promote early detection of premalignant lesions by frequent endoscopic and gynecologic examination.(34) …”

**6. The authors fail to discuss or entertain a number of limitations in their study, namely the reliance on ICD-10 codes (and the inaccuracies therein), the lack of generalization outside a Korean population, the possibility of reporting bias (older subjects, who are more likely to develop cancer, may also be more likely to report insomnia, and age may be a significant confounder -see several references linking increased insomnia to advanced age, for example Miner B, et al. Sleep Med Clin 2017;12(1):31-38.), and how age may be a significant confounder in this analysis (the use of multivariable adjustment alone may not be sufficient to address this confounding).**

***Answer:*** Thank you for your important comments. In the Korean NHIS database, information on ICD-10 diagnosis should be provided when a physician prescribes any examinations or procedures or treatments for the disease. Although severity of insomnia was not available in the NHIS database, we could assume that patients who have the ICD-10 code have insomnia symptoms severe enough to need sleeping medicines or tranquilizers. We revised the paragraph as follows (lines 328-333): “… In addition, reliance on ICD-10 codes and its inherent inaccuracies should be considered. In the Korean NHIS database, information on ICD-10 diagnosis should be provided by a physician who performs any diagnostic tests or procedures or prescribes medications. Although information on the severity of insomnia is lacking, patients with the ICD-10 insomnia code might have insomnia symptoms severe enough to need medications. However, our findings should be confirmed in other ethnicities or countries. …”

**7. Why did the authors not perform a validation analysis on a subset of subjects from their database to ensure their findings could be replicated? They only used 40% of the initial dataset in the initial analysis, they should have data for a validation of a subset (i.e., 20% of the remaining dataset) to validate their initial findings. This is pertinent particularly given some of the unusual associations between insomnia and lower rates of malignancy they have observed.**

***Answer:*** Thank you for your important comments. We understand the need for validation. However, according to institutional regulations, we could only get a 40% simple random sampling database of the subjects who underwent a health-screening examination in 2009. Also, there is a limitation to the access of the KNHIS database due to institutional regulation. That is, we need approval from the institution to get access to the closed server, and we also need to make a reservation to use the database for a limited time. In addition, the location of the server is far from our institution, which makes further analysis difficult. We hope you will please understand our situation.

**8.** **Age is a significant concern regarding confounding. The authors should consider performing a sub-analysis, where they stratify their data by age strata (i.e., every 5 or 10 year age groups as of 2009) and perform multivariable analyses on these subjects to assess insomnia with malignancy. This would better enable them to see if the association has the same value across age groups, They appear to have the dataset size to do this, and it would help alleviate concerns regarding the confounding nature of age in this longitudinal study. Understandably, older age groups would have less follow-up time, but additionally they would be more at risk of malignancy development, so I would be curious to see the results of this type of analysis, and if their findings of an association are maintained.**

***Answer:*** Thank you for your important comments. We completely agree with the reviewer’s concerns. Following the reviewer’s recommendations, we performed a subgroup analysis by age groups (20-39y, 40-59y, 60-79y, and ≥ 80 y). We found the risk of cancer among insomnia patients was significantly different by cancer types (colorectal cancer, liver cancer, lung cancer, bladder cancer, prostate cancer, and breast cancer). We added a new figure (Figure 2) and a supplementary table (Supplementary Table S2), Abstract, the Results and Discussion sessions as follows.

**Abstract (lines 52-54):** “… In the overall study population (N = 3,982,012), the risk for any type of cancer was not different between controls and insomnia patients (adjusted hazard ratio [aHR]: 0.990). However, it was different by age; insomnia increased the risk of any cancer in younger age groups (20-39y and 40-59y, aHR:1.310 and 1.139, respectively) but it significantly decreased the risk in the 60-79y age group (aHR: 0.939). …”

**Results (lines 204-221):** “… ***Subgroup analysis by age strata*** We performed a subgroup analysis by age groups (20-39y, 40-59y, 60-79y, and ≥ 80y). The HR of cancer in insomnia patients of each age group was obtained, and *P* values for interaction was calculated (Figure 2 and Supplementary Table S2). In overall cancer, younger age groups (20-39y and 40-59y) showed significantly higher risk of cancers in patients with insomnia than in controls (aHR [95% CI], 1.310 [1.154-1.487] and 1.139 [1.110-1.180], respectively). In the 60-79y age group, however, we found a s lower aHR for overall cancers in insomnia patients (aHR, 0.939; 95 % CI: 0.916-0.963). Colorectal, liver, lung, thyroid, bladder, prostate, and breast cancers showed significant interaction of age with the association between insomnia and the cancer risks (*P* for interaction < 0.05). Ovarian cancer showed higher aHR in the age 20-39y group (aHR 1.854, 95 % CI: 1.110-3.096). In the 40-59y age group, many cancers such as liver, pancreatic, oral, esophageal, gallbladder, renal, bladder, and male prostate cancer showed higher risk in insomnia patients than in controls (Table 4). In the 60-79y age group, nerve cancer, multiple myeloma, and leukemia showed a higher risk (aHR [95 % CI], 1.231 [1.041-1.455], 1.242 [1.017-1.516], 1.256 [1.051-1.503], respectively), while the risk of stomach, colorectal, and female breast cancers was lower in insomnia patients (aHR [95 % CI], 0.903 [0.848-0.962], 0.843 [0.795-0.893] and 0.809 [0.709-0.924], respectively.) …”

Discussion (lines 310-318): “… Age is an important confounding factor. Thus, we also performed subgroup analyses by age groups. Interestingly, the risk of cancer in patients with insomnia revealed differences according to the type of cancer (Supplementary Table S2). In younger age groups (20-39y and 40-59y), the risk of overall cancers was higher in patients with insomnia (aHR [95% CI], 1.310 [1.154-1.487] and 1.139 [1.110-1.180], respectively), whereas it was significantly lower in the 60-79y age group (aHR, 0.939; 95 % CI: 0.916-0.963), especially in cases of colorectal, stomach, and female breast cancers (Figure 2). As colorectal cancer is a typical cancer that has a precancerous lesion and removal of adenomatous precursors can result in prevention of cancer progression in this age group,(35) health behaviors could have affected our findings …”

**Supplementary Table S2.** Risk of cancers according to insomnia stratified by age group.

|  |  |  | **Age Group (years)** |  |  |
| --- | --- | --- | --- | --- | --- |
| **Cancer Type** | **20-39** | **40-59** | **60-79** | **≥80** | ***p* for interaction** |
|  |  |  |  |  |  |
| All Cancer | **1.310(1.154,1.487)** | **1.139(1.100,1.180)** | **0.939(0.916,0.963)** | 0.991(0.907,1.083) | **<.0001** |
| Stomach | 0.553(0.276,1.106) | 1.024(0.925,1.132) | **0.903(0.848,0.962)** | 1.027(0.825,1.277) | 0.0660 |
| Colorectal | 1.346(0.927,1.954) | 1.074(0.986,1.17) | **0.843(0.795,0.893)** | 1.091(0.902,1.320) | **<.0001** |
| Liver | 1.200(0.622,2.313) | **1.283(1.146,1.437)** | 1.050(0.975,1.130) | 0.912(0.699,1.190) | **0.0138** |
| Pancreatic | 0.685(0.256,1.831) | **1.160(1.014,1.327)** | 0.946(0.872,1.027) | 0.985(0.724,1.340) | 0.0706 |
| Lung | 2.079(1.175,3.680) | **1.354(1.212,1.513)** | 1.087(1.027,1.151) | 0.932(0.761,1.142) | **0.0002** |
| Thyroid | **1.218(1.015,1.462)** | **1.108(1.032,1.189)** | 1.013(0.916,1.120) | **2.293(1.322,3.975)** | **0.0137** |
| Lymphoma | 0.978(0.406,2.360) | 1.103(0.873,1.393) | 1.081(0.925,1.264) | 1.159(0.646,2.078) | 0.9895 |
| Oral | 1.715(0.639,4.603) | **1.487(1.148,1.927)** | 0.990(0.807,1.215) | 0.955(0.458,1.995) | 0.0810 |
| Esophagus | . | **1.504(1.062,2.131)** | 1.13(0.931,1.372) | 1.264(0.626,2.552) | 0.5689 |
| Gallbladder | . | **1.545(1.128,2.116)** | 1.011(0.859,1.191) | 0.760(0.419,1.378) | 0.0732 |
| Biliary | . | 1.113(0.848,1.460) | 1.086(0.96,1.229) | 1.006(0.678,1.494) | 0.9759 |
| Laryngeal | . | 1.529(0.966,2.418) | 0.98(0.741,1.295) | 1.356(0.469,3.922) | 0.4256 |
| Renal | 0.987(0.409,2.380) | **1.276(1.032,1.578)** | 1.16(0.994,1.354) | 0.639(0.278,1.473) | 0.4234 |
| Bladder | 1.932(0.617,6.046) | **1.340(1.052,1.707)** | 0.936(0.825,1.063) | 0.746(0.486,1.145) | **0.0210** |
| Nerves | 0.866(0.278,2.697) | 1.234(0.957,1.592) | **1.231(1.041,1.455)** | 0.629(0.305,1.297) | 0.3194 |
| Multiple myeloma | 2.913(0.715,11.874) | 0.857(0.555,1.323) | **1.242(1.017,1.516)** | 0.732(0.224,2.400) | 0.2082 |
| Leukemia | 1.122(0.419,3.004) | 1.135(0.843,1.529) | **1.256(1.051,1.503)** | 1.379(0.702,2.711) | 0.9233 |
| Skin | 2.964(0.726,12.093) | 1.026(0.562,1.874) | 0.805(0.552,1.174) | 0.306(0.041,2.269) | 0.2222 |
| In men | |  |  |  |  |
| Prostate | . | **1.439(1.224,1.693)** | 1.059(0.984,1.139) | 1.184(0.915,1.533) | **0.0083** |
| Testicular | 0.989(0.138,7.075) | 0.916(0.226,3.712) | 1.269(0.687,2.344) | 4.071(0.369,44.879) | 0.7589 |
| In women | |  |  |  |  |
| Breast | 0.870(0.611,1.240) | 1.066(0.977,1.163) | 0.809(0.709,0.924) | 1.085(0.514,2.289) | **0.0072** |
| Cervical | 0.957(0.496,1.848) | 0.958(0.759,1.209) | 0.964(0.770,1.206) | 0.875(0.344,2.222) | 0.9979 |
| Ovarian | **1.854(1.110,3.096)** | 0.937(0.763,1.149) | 0.923(0.753,1.133) | 0.713(0.283,1.792) | 0.0753 |
| Corpus | 1.154(0.477,2.792) | 0.986(0.775,1.256) | 0.963(0.712,1.305) | 2.992(0.922,9.715) | 0.3230 |

Adjusted for sex, low income, smoking, alcohol consumption, diabetes, hypertension, dyslipidemia and body mass index. aHR, adjusted hazard ratio; CI, confidence interval. Bold style indicates statistical significance.

**Figure 2. Effect of insomnia on the risk of some cancers stratified by age group.** Results of all cancer types are presented in Supplementary Table S2. aHR, adjusted hazard ratio; CI, confidence interval. Thick lines and bold style indicate statistical significance (*p* < 0.05)

*The authors really appreciated your kind and helpful comments. The revisions based on these comments improved the accuracy and the quality of our manuscript. Thank you again.*

Kichul Yoon, M.D.

Cheol Min Shin, M.D., Ph.D.

**Reviewer #2**

Insomnia is the most common sleep disorder and has been shown to be associated with an increased risk for numerous disorders, including cardiovascular, neurological and psychiatric disorders. As such, it merits particular attention in the research community. Yoon et al. describe the association between insomnia in various types of cancer in the Republic of Korea between 2009 and 2018. The authors show that insomnia is associated with an increased risk for several cancers, but also decreased risk for some types of cancer, including colorectal cancer. Due to the inconclusive nature of the connection between insomnia and various types of cancer, this study presents a valuable contribution to the existing literature and understanding behind this connection. While the article is easy to understand and fluent to read, there are several suggestions that, in my opinion, would make the article more comprehensive and novel, thus suitable for publication.

**Abstract:**

**1. Please write exact P values in the abstract where possible.**

***Answer:*** Thank you for your important comments. We added specific *P-*values to the abstract.

**2. Please be slightly more specific (most important/reliable findings) in the conclusion of the abstract.**

***Answer:*** Thank you for pointing this out. Following the reviewer’s recommendation, we revised the conclusion of the abstract as follows: “… **Conclusion:** Insomnia is associated with an increased or decreased risk of some cancers, depending on cancer age, cancer type and sex. …”

**Introduction:**

**3. Please add very brief paragraph about the health implications of chronic insomnia after the first paragraph to fluently transition to the second paragraph about the association between insomnia and cancer.**

***Answer:*** Thank you for the important comments. We added the following paragraph (line numbers are of 'Clean Copy' version of manuscript;) (lines 74-76): “… The association between insomnia and increased risk of various health condition such as depression, dementia, nonalcoholic fatty liver disease, hypertension and cardiovascular disease has been suggested.(3) …”

**4. In the second paragraph, please add more background on why the results associating insomnia and cancer are inconsistent (and what cancers in particular) citing relevant literature.**

***Answer:*** Thank you for your accurate comments. Following the reviewer’s recommendation, we provided a relatively recent systemic review for the statement. In the metaanalysis, 4 of the 8 included epidemiologic studies showed no relationship between insomnia and the risk of cancer. In contrast, four other reports showed a higher risk of cancer among study participants who suffered from insomnia. For example, breast cancer was shown to be associated with insomnia in two studies, but another study reported most sleep characteristics including hours of sleep per night did not correlate with the cancer risk. We added this in the Introduction as follows (lines 83-90): “… A recent meta-analysis showed that there was a 24 % overall increase of cancer risk in insomnia patients.(2) However, according to a systemic review and metaanalysis of previous epidemiologic studies, the results were inconsistent in terms of the risk for any kind of cancers and insomnia. Four of the 8 epidemiologic studies showed no relationship between insomnia and the risk of cancer. In contrast, four other reports showed a higher risk of cancer among study participants who suffered from insomnia.(2) In particular, breast cancer showed positive association between insomnia and cancer in two studies.(8, 9) In contrast, another study with more specific analyses of sleep pattern reported most sleep characteristics including hours of sleep per night did not correlate with breast cancer risk.(10) …”

**5. In the last paragraph, please clearly explain why your study is different compared to already published studies and how it may add value to the field (apart from being another group of patients).**

***Answer:*** Thank you for your important comments. We think the most important value of our study is the large study population with reliable national health insurance service data. We emphasized this perspective as follows (line 97): “… The purpose of this nationwide, large-scale, retrospective cohort study, compared with previous reports, was to show the correlation between various types of cancer and insomnia, based on information provided by the Korea National Health Insurance Service (KNHIS) database. …”

Methods:

**6. Please explain the reasoning behind including 40 % and not all of the patients.**

***Answer:*** Thank you for your important comments. Access to the Korean National Health Insurance Service database is limited due to institutional regulations. We could only get a 40% simple random sampling database of the subjects who underwent a health-screening examination in 2009. We need institutional approval to gain access to the closed server, and we also need to make a reservation to use the database, for only a limited time. In addition, the location of the server is far from our institution which makes further analyses difficult. We hope you will please understand our situation.

**7. Please elaborate whether patients with all cancer stages were included and whether you grouped them in any way, which might add significant value to the literature. I would suggest grouping patients by either cancer stage (early/late…) and/or by age group/sex which would add another novelty factor to your research.**

***Answer:*** Thank you for your important comments. We agree with you that cancer stage would be valuable information for our research. However, the Korea National Health Insurance Service (KNHIS) database does not collect cancer histology or stage data; thus, we could not get this information. We addressed this as a limitation as follows (lines 325-326): “… Second, we had limited use of the KNHIS claims database; thus, data on sedative-hypnotics and antidepressants could not be analyzed further. Additionally, the KNHIS claims database does not collect data on cancer stage or histology. …” We hope you will please understand our situation.

**8. Please explain the sleep history of these patients prior to inclusion if possible since presence/duration of insomnia/other sleep disorders might be an important factor in cancer development. I would suggest grouping patients by insomnia severity and duration to see whether there is any association with different types/stages of cancer, which would be another differentiating factor for your study.**

***Answer:*** Thank you for your important comments. The information on insomnia duration and severity can be very helpful towards the validation of our results. Our database on the diagnosis of insomnia is based on ICD-10 codes, which are needed when a physician prescribes a medication for the disease. Although we do not have questionnaire or other data to evaluate the severity of insomnia, we could assume that patients who have the ICD code have severe enough symptoms to be prescribed medicine. We briefly addressed your concerns as a limitation as follows (lines 328-333): “… In addition, reliance on ICD-10 codes and its inherent inaccuracies should be considered. In the Korean NHIS database, information on ICD-10 diagnosis should be provided by a physician who performs any diagnostic tests or procedures or prescribes medications. Although information on the severity of insomnia is lacking, patients with the ICD-10 insomnia code might have insomnia symptoms severe enough to need medications. However, our findings should be confirmed in other ethnicities and countries. …”

**9. Please elaborate whether all patients had insomnia during the whole period (2009-2018), how their insomnia was followed and whether patients with cured insomnia between 2009 and 2018 were still included in the study till the very end.**

***Answer:*** Thank you for your helpful comments. We defined having insomnia as having the diagnosis code F510 (nonorganic insomnia) or G470 (insomnia) within one year (window period) of 2009. Because the definition of ‘duration’ of an individual insomnia diagnosis is not easy to confirm and immortal time bias could be a concern if the window period is changed individually, we applied the above definition.

**Results:**

**10. Please elaborate on why subjects with newly diagnosed cancer within one year of screening examination were excluded.**

***Answer:*** Thank you for your important comments. We tried to exclude undiagnosed concurrent cancer at the time of the insomnia diagnosis. Thank you!

**Discussion:**

**11. Please elaborate in the discussion whether your findings could be influenced by the region you studied since some cancers are more/less common in the Asian population in general.**

***Answer:*** Thank you for your important comments. Studies specifically on the general Asian population are scarce, but there are several cohort and case-control studies from Taiwan. We referred to those studies and added paragraphs as follows:

(line 234): “… A previous study found that those with fewer than 6 hours of sleep per night had markedly increased risk of colorectal adenomas compared with subjects that slept 7 hours or more,(14) and a Taiwanese study showing higher HR of colon cancer in insomnia patients.(11) Another nested case-control study found the risk of colorectal cancer was higher in patients with insomnia.(15) …”

(line 260): “… The result was not consistent with a previous Taiwanese case-control study, which reported higher risk of the cancer among patients with insomnia.(11) …”

(line 267): “… In contrast, a study on a Taiwanese sample reported most sleep characteristics did not correlate with breast cancer risk,(10) which is consistent with our findings. …”

Figures:

**12. I suggest adding a figure (e.g., Forrest plot) that will highlight the most important results in a visually appealing way, in addition to the tables where you can write all the results.**

***Answer:*** Thank you for pointing this out. We decided to add a figure (Forrest plot) on risk of cancers according to insomnia stratified by age group, which we further analyzed for this revision, as Fig. 2 (Results of all cancers are presented as a Supplementary Table S2.) Please refer to our novel finding on the result section ' Subgroup analysis by age strata' line(204-221).

**Figure 2. Effect of insomnia on the risk of some cancers stratified by age group.** Results of all cancer types are presented in Supplementary Table S2. aHR, adjusted hazard ratio; CI, confidence interval. Thick lines and bold style indicate statistical significance (*p* < 0.05)

These suggestions are likely to make the article more comprehensive and novel and I feel they are necessary for this article to be accepted for publication.

*The authors really appreciated the reviewer’s kind and accurate comments. The revision based on these comments made this manuscript more accurate and the quality improved. Thank you again.*

Kichul Yoon, M.D.

Cheol Min Shin, M.D., Ph.D.

**Reviewer #3**

Dear authors,

I have now completed the review of the manuscript titled "Risk of Cancer in Patients with Insomnia: Nationwide Retrospective Cohort Study (2009-2018)." In the present study, the authors investigated the association between insomnia and the risk of various cancers using data from the Korean National Health Insurance Service database. The manuscript is interesting and, in general, fair written. I have some minor suggestions before recommending an "accept."

**1. The background section introduced some relevant articles. Please summarize the results with effect sizes.**

***Answer:*** Thank you for pointing this out. We tried to provide effect sizes of the previous studies. However, it seems too complicated to present the effect size for each cancer. Thus, we decided to simply provide the hazard ratio of overall cancer with insomnia for Ref. #8 (Fang HF et al. J Cancer 2015;6:1140-7.) as follows (line 79): “… A nationwide case-control study in Taiwan showed that patients with insomnia exhibited significantly increased risks for overall cancer (adjusted hazard ratio [aHR]: 1.71, 95 % confidence interval [CI]: 1.66-1.77) and many types of cancers (tracheal, nasal, liver, cervical, oral, colon, lymphatic, thyroid, myeloma, prostate, bladder, and kidney).(8) …” Please understand our decision. Thank you!

**2. I suggest authors clarify how other researchers can obtain the original data.**

***Answer:*** Thank you for your important comments. There is a limitation to the access of the Korean National Health Insurance Service database due to institutional regulations. We need institutional approval to gain access to the closed server, and we also need to make a reservation to use the database, for only a limited time.

**3. How many people have F510 (nonorganic insomnia) or G470 (insomnia) each? Could you also compare the rate with previous studies?**

***Answer:*** Thank you for your important comments. We added the data based on each diagnosis code as follows.

**Supplementary Table S1**. Prevalence of ICD-10 code insomnia in the Korean population in the year 2009

|  | Total | Male | Female |
| --- | --- | --- | --- |
| Population | 3982012 | 2198911 | 1783101 |
| Total Insomnia | 134068 (3.37) | 48092 (2.19) | 85976 (4.82) |
| F510 | 53602 | 19700 | 33904 |
| G470 | 91474 | 32339 | 59133 |
| Both | 11008 | 3947 | 7061 |

To date, there are several population-based studies that have used ICD codes for the diagnosis of insomnia.

#1 Chiu et al. 'Insomnia But Not Hypnotics Use Associates with the Risk of Breast Cancer: A Population-Based Matched Cohort study' used ICD-9-CM code.

#2 Fang et al. (Risk of Cancer in Patients with Insomnia, Parasomnia, and Obstructive Sleep Apnea: A Nationwide Nested Case-Control Study) used insomnia (ICD-9-CM codes 307.40, 307.42, 307.44, 307.49, and 780.52).

#3 Lin et al. ‘The association between sleep disorders and the risk of colorectal cancer in patients: A Population-based Nested Case–Control Study.’ In this study, they used ICD-9 code 780.52 (insomnia, unspecified) only.

However, they were either a matched cohort study or a nested case-control study which did not provide the prevalence of insomnia in the general population based on the ICD-9 code. Thank you!

**4. In the ‘Statistical Analysis’ section, the paragraph is quite long but there are no references for selecting your method. Please add two references to the following sentence: Student’s t-test was performed for the comparison of continuous variables and the χ2-test was used for categorical variables [1, 2].**

***Answer:*** Thank you for your important comments. We added the references to the regarding sentences as follows (line 00): “… Student’s t-test was performed for the comparison of continuous variables and the χ2-test was used for categorical variables.(12, 13) …”

*The authors really appreciated the reviewer’s kind and accurate comments. The revision based on these comments made this manuscript more accurate and the quality improved. Thank you again.*

Kichul Yoon, M.D.

Cheol Min Shin, M.D., Ph.D.
