# Supplementary material for: Risk of cancer in patients with insomnia: Nationwide retrospective cohort study (2009–2018)
Source: PLoS One. 2023 Apr 21;18(4):e0284494. doi: 10.1371/journal.pone.0284494 (PMC10121030; doi:10.1371/journal.pone.0284494)
Supplement: Attachment — Submitted filename: Response to Reviewrs_2nd_revision2.docx [file pone.0284494.s007.docx]

March 22^nd^, 2023

Dear Editor:

**RE:** "Risk of Cancer in Patients with Insomnia: Nationwide Retrospective Cohort Study (2009-2018)” (Manuscript ID: PONE-D-22-33830)

coauthored by Kichul Yoon, Jin Hyung Jung, Eun Hyo Jin, Joo Hyun Lim, Seung Joo Kang, Yoon Jin Choi, and Dong Ho Lee

Thank you very much for giving us one more opportunity for revision.

Accurate and kind comments by the reviewer have been addressed in the discussion. We also believe that these comments improved our manuscript. Changes have been made by changing the color to RED in the revised manuscript, tables and figure legends to avoid any confusion.

I anticipate good response.

Thank you!

Sincerely,

Cheol Min Shin, M.D., Ph.D.

Kyungdo Han, Ph.D.

**Reviewer #1**

• The authors have done a good job of responding to my critiques. I find the stratification of insomnia by age to be quite interesting, significantly higher risk in the younger population, but curiously a decreased hazard in the holder age group, and the interaction between age, duration of insomnia, and other associated characteristics (health seeking behavior, hypochondriasis, etc.) is quite interesting.

• I would recommend the authors indicate the limitations of their database as it relates to why they had to use only 40% of the initial cohort, and also why it would be difficult for others to gain access to their data for validation. Per the authors, “according to institutional regulations, we could only get a 40% simple random sampling database of the subjects who underwent a health-screening examination in 2009. Also, there is a limitation to the access of the KNHIS database due to institutional regulation. That is, we need approval from the institution to get access to the closed server, and we also need to make a reservation to use the database for a limited time.”. I suggest paraphrasing this explanation go in the Methods – Study population section.

***Answer:*** Thank you for your important comments that improved our manuscript. Following the reviewer’s recommendation, we revised regarding sentences in the Method - Study population section as follows (line 114, page 6); “… Due to the regulations of the Korean National Health Insurance Sharing Services (NHISS) which limit total sample number and data size, we could only get a 40 % simple random sampling database of the subjects who underwent a health-screening examination in 2009. Also, there is a limitation to the access of the KNHIS database due to institutional regulation. That is, we need approval from the institution to get access to the closed server, and we also need to make a reservation to use the database for a limited time. …”

*The authors really appreciated your kind and helpful comments. The revisions based on these comments improved the accuracy and the quality of our manuscript. Thank you again.*

Kichul Yoon, M.D.

Cheol Min Shin, M.D., Ph.D.

Reviewer #2: I would like to thank the authors for addressing my concerns and I think the article is ready to be accepted.

***Answer:*** Thank you for your important comments that improved our manuscript.

Reviewer #3: All comments have been addressed. Thank you to the authors and editors for considering my opinion on this manuscript.

***Answer:*** Thank you for your important comments that improved our manuscript.
